# Air Pollution Control Policies in China: A Retrospective and Prospects

**DOI:** 10.3390/ijerph13121219

**Published:** 2016-12-09

**Authors:** Yana Jin, Henrik Andersson, Shiqiu Zhang

**Affiliations:** 1 Institute of Environment and Economy (IoEE), College of Environmental Sciences and Engineering, Peking University, Beijing 100044, China; jin.yana@pku.edu.cn; 2 Toulouse School of Economics, University of Toulouse Capitole, 31015 Toulouse CEDEX, France; henrik.andersson@tse-fr.eu

**Keywords:** air pollution, control policy, China

## Abstract

With China’s significant role on pollution emissions and related health damage, deep and up-to-date understanding of China’s air pollution policies is of worldwide relevance. Based on scientific evidence for the evolution of air pollution and the institutional background of environmental governance in China, we examine the development of air pollution control policies from the 1980s and onwards. We show that: (1) The early policies, until 2005, were ineffective at reducing emissions; (2) During 2006–2012, new instruments which interact with political incentives were introduced in the 11th Five-Year Plan, and the national goal of reducing total sulfur dioxide (SO_2_) emissions by 10% was achieved. However, regional compound air pollution problems dominated by fine particulate matter (PM_2.5_) and ground level ozone (O_3_) emerged and worsened; (3) After the winter-long PM_2.5_ episode in eastern China in 2013, air pollution control policies have been experiencing significant changes on multiple fronts. In this work we analyze the different policy changes, the drivers of changes and key factors influencing the effectiveness of policies in these three stages. Lessons derived from the policy evolution have implications for future studies, as well as further reforming the management scheme towards air quality and health risk oriented directions.

## 1. Introduction

Air pollution in China has been very severe, causing huge health damages and social losses for a long time. In the meantime air pollution control polices have over the last couple of decades experienced profound changes, especially a transition from weak to strong implementation. Recently air pollution in China has evolved into an issue of wide and politically prioritized concern. National large-scale polices have been initiated and implemented, such as the Air Pollution Prevention and Control Action Plan (hereinafter the Action Plan) [1]. It is well known that energy (or more precisely coal) is the primary source of air pollution and carbon emissions in China, and the closely related energy and climate polices are also experiencing significant changes.

People who wish to take a closer look beneath the surface of China’s air pollution control policies are immediately faced with difficulties. Research-based information is scattered in several lines of the literature: scientific publications in disciplines such as atmospheric chemistry require some discipline specific expertise of readers. Reviews by experts in these disciplines focus more on air pollution itself, and the policy-relevant discussions provided are broad, and from a national perspective (e.g., [2,3,4]). Environmental governance and law literatures usually discuss Chinese environmental regulation as a whole, seldom discussing air pollution control policies independently (e.g., [5,6,7,8]). Environmental economics studies mostly provide a descriptive background of air pollution control and then focus on individual policies (e.g., [9,10,11]). There are recent Chinese empirical studies (e.g., [12,13,14,15,16]) and some reports on air pollution control policies mentioning the latest developments. However, aside from some short commentaries (e.g., [17]), more detailed, systematic follow-ups are rarely seen.

This paper aims to provide an updated and in more depth review of the Chinese air pollution situation and its related policies from the 1980s and onwards, and to show that air pollution control policies in China are experiencing profound changes. With China’s significant role on air pollution, deep and up-to-date understanding of China’s air pollution policies is of great relevance. Moreover, as they did undergo several significant and different regulatory changes the empirical evidence provides opportunities to learn from these experiences.

The paper is structured as follows: the next two sections first describe the air pollution status and its trends, and then the environmental regulatory system. With these scientific and institutional backgrounds we then describe the three stages of air pollution control polices. We discuss the driving factors for policy changes and conclude by summarizing trends and challenges for the future. It is important to first note that in this review we focus on policies that directly target air pollution. Air pollution is closely related to some other key challenges for China, e.g., climate change, energy and regional cooperation, therefore also related with energy and climate policies. However, during most of the time period which this review covers, the air pollution control policy process in China has been relatively independent of climate or energy policy process. Recently this has changed and with processes being more integrated, a trend that we discuss in Section 5.2.

## 2. Air Pollution Status and Evolution

In China, the major air pollution problems have been mainly attributable to coal burning and industry processes during the early stage of economic development. Recently vehicle emissions have become an increasing problem in urban areas, especially the megacities. A reduction of primary pollutants (primary pollutants are directly emitted from certain sources; secondary pollutants are formed in atmospheric chemistry reactions), e.g., sulfur dioxide (SO_2_), in terms of emission and concentration has been observed, but a more complex, regional air pollution problem dominated by fine particulate matter (PM_2.5_; total suspended particulates (TSP) include all particulate matters (PMs) with different aerodynamic diameters) and ground level ozone (O_3_) has emerged.

### 2.1. Air Pollution Status and Social Impact

Chinese economic growth has been powered by a coal-dominated surging fossil fuel consumption resulting in the highest emission levels for such as carbon dioxide (CO_2_), SO_2_, nitrogen oxides (NO_x_) and primary PM in the world. Regional uneven development concentrates most of the population, economic activities, and emissions in eastern and central China, resulting in an overall severe air pollution situation in these regions, with its city clusters being most heavily affected. The ambient air pollution in China has been estimated by the Global Burden of Disease Study (GBD) to lead to 1.2 million premature deaths from one year’s (2010) exposure in China [18], and in multiple studies to cause annual economic loses equivalent to between 1% and 7% of China’s GDP [12], partially or even totally offsetting China’s current annual GDP growth.

### 2.2. Total Emission, Emission Intensity, Air Quality and Health Damage Trends

**Figure 1:**
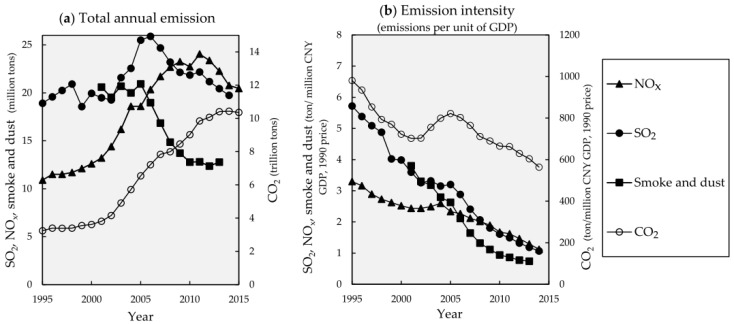
(**a**) Total annual emission; (**b**) Emission intensity of major pollutants and CO_2_ in China, 1995–2014. Data on emissions of SO_2_, smoke and dust, and total suspended particulates (TSP) from National Bureau of Statistics of China (CNBS) [22]; data on CO_2_ emission from global carbon atlases [23,24,25]; emission intensity data is calculated based on the 1990 price in CNY.

**Figure 2:**
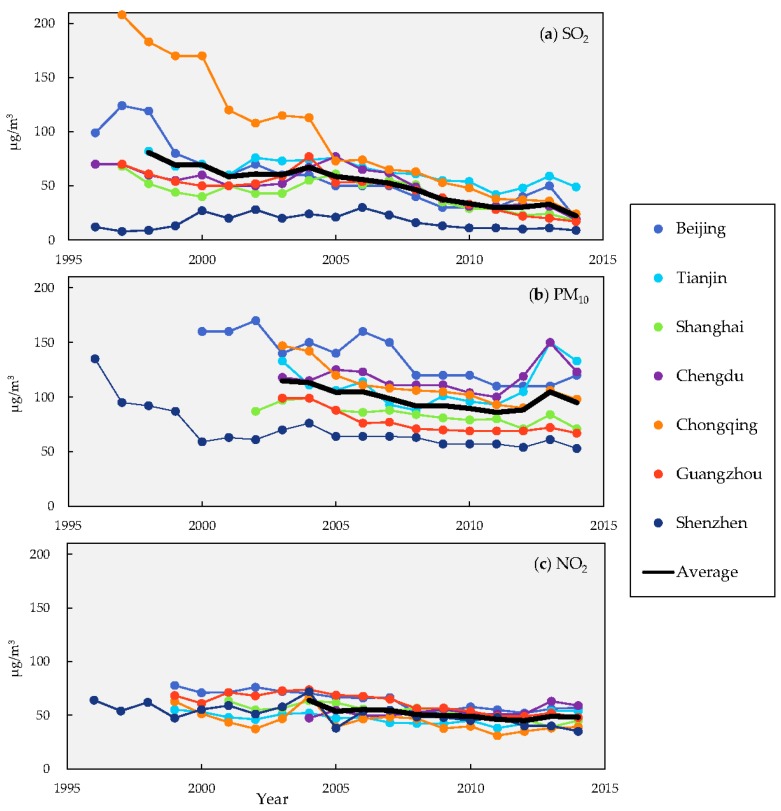
Trends of SO_2_ (**a**); PM_10_ (**b**) and NO_2_ (**c**) annual concentrations of seven China megacities in 1996–2014 [26,27,28,29,30,31,32].

The two, for long regulated, emissions SO_2_ and TSP (including PM_10_, in this paper referred to “smoke and dust” in statistics yearbooks) have passed their peak and are diminishing (Figure 1a), and their concentrations have significantly declined [19,20] (Figure 2a,c). Meanwhile, the less regulated NO_x_ has continued to increase in emission (Figure 1a), with the NO_x_ concentration being stable (Figure 2c and in [21]). For these primary pollutants, their emission intensities indicated by emissions per unit of GDP have declined (Figure 1b).

**Figure 3:**
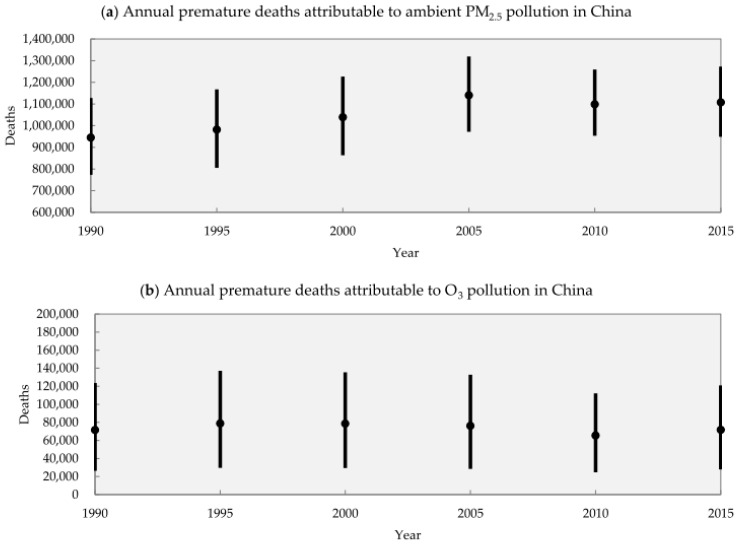
China’s annual premature deaths attributable to PM_2.5_ (**a**) and O_3_ (**b**), 1990–2015. Dots show the estimated base value, bars show lower and upper bound of estimates. Based on data in the Global Burden of Disease Study 2015 [37]. Note the different scales of the vertical axes in (**a**,**b**).

For PM_2.5_ and O_3_ concentration, the situation is worsening [33]. Moreover, regional pollution problems are becoming significant, especially when weather conditions are not good for pollutant diffusion and degradation. Vast regions, even the whole eastern and central China, can sometimes be under very high concentration of PM_2.5_ and O_3_ [34,35]. Ambient air pollution in the form of PM_2.5_ and O_3_ leads to more significant public health effects compared to that of primary pollutants [18,36]. The GBD estimated China’s annual premature deaths attributable to PM_2.5_ and O_3_ have remained high, summing average values of around 1 to 1.2 million deaths from 1990 to 2015 (Figure 3).

### 2.3. Atmospheric Chemistry Explanation and Emission Source Changes

The very high levels and spreading trend of PM_2.5_ and O_3_ are the result of an expanding secondary particle production atmospheric chemical reaction process, with increasing amounts of reactants and accelerated reaction rates due to the higher oxidation capacity. A concise scientific explanation for this is “air pollution complex” (or compound/complicated/complex pollution, a concept formally proposed and defined by Tang in 1997, and was further explained and developed in [38]).

O_3_ is formed by the reactions of NO_x_ and Volatile Organic Compounds (VOCs) under solar radiation. Areas of elevated fine particulate concentrations can also form downwind of the precursor source areas if there is considerable movement of air. More importantly, atmospheric oxidation capacities are enhanced by increasing O_3_ concentrations. Thus, SO_2_, NO_x_, and VOCs will be transformed into PM_2.5_ more efficiently where O_3_ concentrations are higher due to increased rates of oxidation.

This motivates a further look at the changes of emission sources by, as generally defined, three stages: (1) In 1970–1990, the dominant contributing sources were big and small coal burning stoves widely used in power plants, industry, utilities and households. Coal smoke mainly contains SO_2_, TSP, but also NO_X_ and carbon monoxide (CO). Other sources, such as dust from construction site mainly consisting of primary PMs, contributed less; (2) During the transition period 1990–2000 the growing number of vehicles, mainly in mega cities contributed a lot to the increase in NO_x_ and VOCs (for example, vehicles were found to contribute to about 30%–56% of total Pearl River Delta regional NO_x_ and Non-Methane VOC emissions [39,40]) which were added to the previous problems [38]. As a result, secondary PM_2.5_, and O_3_ increased, the “air pollution complex” took shape, with various primary and secondary pollutants involved in the atmospheric chemical reactions; (3) From 2000 to the present, the compound air pollution is ingrained in the megacities and spreading to the regional level, corresponding to the trends that vehicle ownership continues to soar and expand to surrounding areas of the cities. On the national level coal burning is still dominant in contributing to the primary pollutant emissions [41], whereas for megacities local specific source apportionments show that none of the contributions from various sectors such as coal burning, vehicles, construction site dust, industrial processes, are possibly ignorable [42]. Moreover, regional transmission also plays an important role [43].

In China the air pollution situation is severe, widely distributed, and the atmospheric chemical reactions complex. These characteristics indicate no easy solution and that any effective control strategy has to target all the major pollutants from different sources, and control measures have to be jointly designed and wisely combined. It is evident that China therefore faces different challenges than developed countries, which experienced and solved different phases of air pollution for nearly a century.

## 3. Environmental Regulatory System

**Figure 4:**
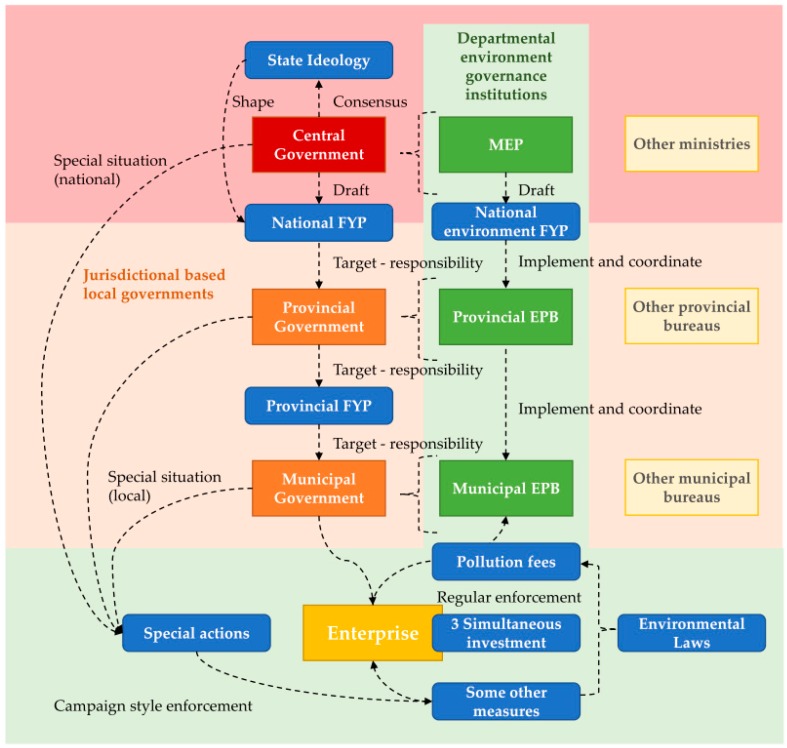
Environmental governance in China.

The institutional composition and dynamics of the environmental regulatory system in China is key to understand the air pollution control policies. In this section we rely on accumulated knowledge from the governance literature (e.g., [6,8,44,45]) to lay out the institutional and environmental governance foundation for later sections. The environment regulatory system in China is viewed as a very comprehensive regime. However in most cases implementation and enforcement of environmental regulations have been weak and questionable during the past decades. As an illustrative summary, Figure 4 (discussed in more detail later) shows the institutional structures and the channels through which the policies and government departments work.

### 3.1. Government Institutions Foretell the Implementation Difficulty

Chinese governance is departmental-regional fragmented. The regional dimension spreads from central government (the red area in Figure 4) to jurisdictional-based local governments. The departmental dimension contains each ministry in the central government and the same functional agency in local government layers (e.g., from the Ministry of Environment (MEP) to local environmental protection bureaus (EPB), the green area in Figure 4). Among the local officials, the chief leaders of the Party Committee and the Government (e.g., at municipal level, party secretary, vice-party secretary, mayor and vice-mayor) are the ones in charge of public affairs within their geographic jurisdiction. They also manage the allocation of personnel and money among different bureaus in the local government (orange area in Figure 4). As they are largely promotion driven, their limited attention and the tradeoffs in resource allocation are naturally influenced by the incentives given by the promotion mechanisms.

Promotion of local officials in China is a complicated “cadre evaluation” process in the Five-Year-Plan (FYP) framework (see, e.g., [8]). The FYPs are a cycle process starting from the release of a series of social and economic development initiatives by the central government, among which many goals are “administratively subcontracted” [44] to local governments. The bureaucratic personnel are supposed to fulfil these directives and be evaluated on these upper assigned tasks and eventually will be promoted or not. Some key characteristics of this process are: (1) the ones getting higher rankings are more likely to be promoted; (2) the strongest promotion incentive is concentrated on the local main leaders; and (3) though subcontracted tasks are numerous, covering most public areas, only economic development (e.g., GDP) is tracked and veto-track factors largely determine the evaluation ranking. A veto means that if the target is not fulfilled, there will definite be no chance for promotion, one example was birth control targets.

Environmental protection is among the highly subcontracted jurisdictional obligations. However, for a long time it was not included in the two emphasized tracks of factors in the promotion assessment. Therefore the difficulties in its implementation are largely inherent and only in relatively rare cases (shown as the special situations in Figure 4) will the local main leaders’ attention be temporarily drawn to environmental issues, such as when certain environmental problems become severe and urgent to the extent as to cause a veto, or somehow higher level of government mandates them to pay attention. In “normal” situation EPBs work “alone” on local environment issues. Lack of attention is compounded by the lack of capacity in EPBs, they hardly have a sufficient fiscal budget and personnel for monitoring and enforcement.

Weak implementation as the overall result of the constraints is not destined to persist though. When the rules of promotion are changed and the veto track factors begin to include environmental ones, local main leaders have to pay attention. When these factors start being measured in a way that cannot easily be hidden by other flexible measures (e.g., data fabrication, see, e.g., [46]), local main leaders have to really implement the regulations. Further, when strong political pressure from the central government is perceived by local governments, the latter may even “over implement” and “innovatively implement”.

### 3.2. What Are the Environmental Policies?

China’s environmental policy system generally consists of broadly five series of polices (shown as blue blocks in Figure 4), systematically intertwined in the departmental-regional government described in Section 3.1. They are: (1) environmental laws, rules and standards; (2) national plans in the FYP framework; (3) ten specific regulatory measures; (4) special actions outside the FYP framework; and (5) environment-related state ideologies. As aforementioned, apart from environmental policies, energy and climate policies are also highly relevant to air pollution, but for a long time they were part of another relatively independent policy system in the context of climate changes and energy security (for a review of this track of policies, see, e.g., [47]). Here we focus on environmental policies.

#### 3.2.1. Environmental Laws and Standards

China has many environmental laws at different legislative levels. In 1978, the Third Chinese Constitution was issued with inclusion of an environmental commission. The Environmental Protection Law was issued on a trial basis in 1979 and then formally in 1989. Subsequently China has issued around 30 laws related to the environment (for example, the Law on the Prevention and Control of Atmospheric Pollution), hundreds of administrative rules, and even more detailed standards. Wang has reviewed environment law in China and summarized them as covering every aspect of environment management, but to have low legislative quality, too many principles, being very basic and difficult to actually enforce [48].

However, the various emission and environment quality standards in the environmental laws do provide a quantitative summary of the development of China’s pollution control targets and measures. Take air pollution control as an example, Table 1 lists representative standards on coal fired power plants, coal-burning boilers, vehicles, and on air quality. From this table it becomes evident that these standards in China were established early, even in the 1980s, and then were fixed or even loosened (the air quality standard) around 2000. After a dozen of years (note that the PM_2.5_ crisis happened in the winter between 2012 and 2013), these standards have been updated and significantly strengthened. The current standards are largely in line with international ones and for power plants even more stringent than those in developed countries such as the U.S. and Japan.

#### 3.2.2. National Environmental Plans within the FYPs

National environmental planning within the FYPs framework started from scratch and now dominates environmental governance in China [6]. Generally the more emphasized public areas will have concrete chapters in the nationwide FYP, their goals will be more quantitatively articulated and be supported with more focused FYPs, even with industry specific FYPs (e.g., the 11th FYP on “National environment protection” and on “Coal fired power plants SO_2_ emission control” [50]). Among the many public area targets, the solid type is “mandatory” targets. They are guaranteed to be attained and implemented in advance [51]. These targets are decomposed and allocated to local governments through the target responsibility agreement system and integrated to veto track factors in cadre evaluation. Environmental protection entered the 6th FYP as an independent chapter and became more specific and concrete in later FYPs [52]. The 11th FYP (2006–2010) is the milestone in which for the first time several quantitative environmental and energy targets became mandatory [53].

#### 3.2.3. Ten Specific Conventional Regulatory Measures

During the past decades, ten specific conventional regulatory measures have been solidified. These measures are often referred to as “Old Three, New Five, Target Response and Total Emission Control” (Old Three refers to 1 to 3 of the list, and were solidified between 1972 and 1979, New Five refers to 4 to 8 and are solidified between 1980 and 1989. “Lao San Xiang, Xin Wuxiang, Mu Biao Ze Ren Zhi, Zong Liang Kong Zhi” in Chinese) in China’s environmental management policy context [54,55]. Generally when talking about environmental regulatory implementation in China, it is one or several of these measures being used:

1. Construction project environmental impact assessment (EIA): Construction projects must conduct EIAs before their construction. In 2003 this measure was expanded to including planning EIAs.
2. “Three Simultaneously” (3S): Installations for the prevention and control of pollution at a construction project must be designed, built and commissioned together with the principal part of the project.
3. Pollution fees: When discharging pollutants in excess of the discharge standards, a fee shall be paid for excessive discharge. In 2003 pollution fees became collectable on all the discharges and no longer restricted to excessive discharge.
4. Comprehensive quantitative evaluation of urban environment: A weighted grading system with indicators covering all aspects of environment protection. Annual grading results with only ranking information of about a hundred cities are made public.
5. Pollutant discharge permit: Polluting units shall discharge pollutants in accordance with their permits. Certificates of pollutant discharge permit shall be applied at EPB, in which major pollutants and information of discharge pipes are specified.
6. Undertake treatment within a prescribed limit of time: The legislative institutions make decisions that require companies, identified as being involved in heavy pollution, to dispose of the pollution source within a time limit, to reach the specified disposal demanded.
7. Centralized pollution control: Treatment facilities for polluting units within a limited area are jointly planed, designed and operated, as an additional mechanism to the conventional independently performed way.
8. Cleaner production: Liable enterprises shall monitor resource consumption and generation of wastes in production, and conduct cleaner production audits and report to the relevant administrative departments in the local government.
9. Environmental protection target responsibility system: Local governments at or above the county level are responsible for environmental protection in their jurisdiction and the responsible persons will be assessed on this in cadre evaluation.
10. Total emission control: Within a limited time and area, aggregated emission quotas less than a certain cap are allocated to local governments/targeted enterprises. Early trials were in the “Acid Rain and SO_2_ Control Zone” [56]. Later this became a nationwide measure.

#### 3.2.4. Special Actions outside the FYPs

China is able to quickly respond to all kinds of “situations” (e.g., crisis, special needs, and environmental accidents) [6,57]. When the environment becomes deteriorated with the impact acute to a certain level, special actions can temporarily relieve the ineffectiveness of regular policies. Usually they are local, short-term administrative measures at any costs [58]. Sometimes, after a certain situation is subsided, parts of these measures will be adopted at regional or national level and even be integrated in later FYPs [6]. Some examples of special actions are the Environmental Law Enforcement Inspection, believed to temporarily draw local main leaders’ attention to environment protection [44], EIA Storm in order to partially rectify the common cases of “construction first and EIA later” [59], and some radical actions in Beijing Blue Sky project when preparing for the 2008 Olympics and the Action Plan as triggered by the PM_2.5_ crisis.

#### 3.2.5. Environment Related State Ideologies

“State ideologies” in China are soft policies in the broad sense. On the surface they look like slogans whereas they can reflect the central political consensus on some social issues and guide the national planning. In terms of the possibility in deriving some concrete policies they may have very different results. World-known and thoroughly implemented state ideologies are “Focus on Economic Construction” and “Family Planning”. Historic experience shows that a dozen of environment-related state ideologies (“Protect the Environment”, “Sustainable Development”, “Cleaner Production”, “Circular Economy”, “Green GDP”, “Harmonious Society”, “Scientific Development”, “Beautiful China”, “Low-Carbon Economy” and “Resource-Conserving and Environmentally Friendly Society”. The latest one is “Ecological Civilization”) largely accomplished their mission as slogans, but from their official interpretations, the “win-win” theme of environment protection and economy development has become clearer over time [6].

### 3.3. An Illustrative Summary

We designed Figure 4 aiming to display the institutional structures, dynamics and policies in a systematic way. However, it does not indicate a rigid environmental governance system, and does not show some past and ongoing changes in this system. For example, the administrative rank of the environment protection department in the central government has been increasing, from a working group in the 1970s all the way up to a ministry status in 2008. The Environment Protection Law, after remaining unchanged for 25 years, has been amended in 2014 and some of the new law articles are regarded as unprecedentedly strict [60]. Non-governmental actors, not shown in Figure 4, have been granted the legal power to claim environmental rights [61], hence their role is increasing.

## 4. Three Stages of Air Pollution Control Policies in China

### 4.1. Until 2005, before the 11th Five-Year Plan

Based on previous trials focusing on eliminating industrial smoke and dust, the basic system of air pollution control was established in the Law on the Prevention and Control of Atmospheric Pollution, issued in 1987. The system can be summarized as: (1) local governments are responsible for air pollution control within their jurisdiction; (2) a concentration on point sources, especially the big industrial coal burning units; (3) measures include standards of emission concentrations at the stacks, pollution fees, guidance of end-of-pipe treatment techniques and the corresponding 3S requirements; and (4) if the law is violated, fines with the maximum capped at 500,000 RMB (exchange rate: 1 USD = 8.28 RMB in 2000) shall be applied. Usually this is at most a small percentage of the fixed investment. Though this basic system experienced a string of small changes, it saw no substantial reform in the subsequent decades.

In terms of the regulatory effects, we focus on the two measures related with observable physical outcomes, the 3S and pollution fees. The reality of implementation of the 3S was puzzling. Its implementation rate increased from 87% in 1994 to 98% in 1999, whereas the ratio of investment in 3S over total fixed assets kept around 5‰. If both were true it would imply that new fixed investment went to less polluting industries, which is not supported by the official statistics [58]:

(1) $$\begin{array}{c} \frac{{Invest}_{3s}}{Total\;invest}=\frac{{Total\;invest}_{dirty}\times implementation\;rate\;of\;3S\times \beta }{{Total\;invest}_{dirty}+{Total\;invest}_{clean}}\\ =\frac{implementation\;rate\;of\;3S\times \beta }{1+\frac{{Total\;invest}_{clean}}{{Total\;invest}_{dirty}}}=5‰ \end{array}$$

The pollution fee was a small levy on enterprises. Theoretically, its social efficient level equals the marginal damage cost. However, in most industries pollution fee levels were much lower, even lower than average abatement costs (e.g., in 2005 the pollution fee for SO_2_ was 0.63 yuan/kg, while the average abatement costs of SO_2_ in coal fired power plants were between 4 to 6 yuan/kg [62]). “Deal enforcement” [58] was in some cases the result, far from the original policy intention, with enterprises actively paying the fee but not abating, and the EPBs actively collecting this fee to support their own daily operations.

#### 4.1.1. Some Fundamental Flaws

Looking back on what actually happened before 2005, air pollution, severe though, was not regarded as an urgent social problem. The nature of the management regime was not so much a broad air pollution prevention and control, as a limited project management on how enterprises do emission discharge. It did have positive regulatory effects, but to a larger extent became a fetter on subsequent policy adjustments. The limitations are clear:

1. The general principles of environmental rights and interests are absent. It lacks the concept that the atmospheric environmental capacity is natural capital, is scarce and has value. It does not have an ultimate regulatory aim to protect human health and enhance social welfare.
2. Inefficiencies are present. Air pollutants transmit and transform but the management is jurisdiction-based, therefore “leakage” and lack of regional coordination are predestined to happen. Campaign style regulations incur high administrative cost with transient effects. Pollution fees are too low compared to the efficient level.
3. Monitoring capacity as a basic regulatory element is not created, therefore all policy instruments designed based on the emission situation become ineffective. Estimated data with information on inputs or experiences hardly reflect the real emission situation (see, e.g., [63]).
4. Credibility of the law and regulations is seriously challenged. The cost of breaking environmental laws is strikingly low [64]. Legal liability theoretically exists, but judicial latitude of discretion is huge due to the too vague laws [48]. Campaign style regulations always come and go in haste. All this preserves an expectation that they are and will be tacitly permitted to be incompletely implemented.

#### 4.1.2. Special Case 1: Beijing Fought a Lone Battle

The Chinese central government is located in Beijing, where the air quality has long been notoriously bad. Unlike that in other places, in Beijing air pollution control was a political task with support from the central fiscal budget. As early as 1998, with direct instructions from the central leadership, air pollution control in Beijing became the top priority in environment management in China [65]. Leading the rest of China, Beijing adopted measures such as to substitute coal by clean fuels in certain zones, to apply desulfurization, dust collection and low NO_x_ combustion techniques in power plants, to increase green land covering and control dusts from construction sites, and a series of measures on vehicles including emission standards for new vehicles, high quality fuel supply, detection of in-use vehicles and early retirement of heavy-duty and old vehicles [66]. However, due to the surge in vehicles and the transported emissions from industrializing neighbors [67], the specific efforts of Beijing were not enough to bring about any significant air quality improvement. These efforts were not totally in vain, though, by providing important evidence; vehicle exhausts control, among all the measures, was found to contribute the most to concentration abatement potential of NO_x_ and PM_2.5_ in Beijing [68].

### 4.2. 2006–2012, in the 11th Five-Year Plan and Early 12th Five-Year Plan

Based on what happened until 2005 many studies, as summarized in [63], predicted that without fundamental institutional reform in China the “implementation gap” in environmental governance could hardly be overcome. However, in the 11th FYP and early 12th FYP (2006–2012) China did implement significant policies on air pollution, given an institutional arrangement largely unchanged. The policies were the “total emission control on SO_2_” and the closely relevant “energy saving” policy. For SO_2_ and chemical oxygen demand (COD, a measure of organic compounds in water) a 10% reduction from the 2005 level was set as the national target. On energy saving, the target was a 20% reduction of energy consumption per unit of GDP from the 2005 level. National targets were decomposed among provinces, which led all the subordinate municipal governments to fulfill the upper assigned target. In the 12th FYP (2011–2015) these policies were maintained and extended.

It should be noted that the national total control policies were initiated in the 9th FYP and further specified in the 10th FYP but back then the implementation and results were failures (see, e.g., [10]). In the 11th FYP, the implementation became remarkable and the targets were achieved. However the air quality improvements were insignificant. Then three key questions arise: (1) Why did the local governments implement the policies? (2) How did they implement the policies in different sectors and industries? (3) Why the efforts are ineffective and inefficient in improving air quality? These three questions are discussed in the following sections.

#### 4.2.1. Why Did Local Governments Implement?

Various studies, as summarized in [69], seem to reach a consensus that one key element for the successful implementation of total control policies was that each province’s target became unprecedentedly “mandatory” and was linked to the veto track factors in cadre evaluation of local main leaders. If the annual target were not met, then the local leaders were ordered to rectify within a time limit. If the five years’ target failed, they would get a veto. This shift provided incentives for local leaders to pay attention to these environmental targets. However, this could not ensure them to really do something if emission data could still be relatively easily adjusted. That was exactly what happened in 2006 when total SO_2_ and COD emissions increased 1.9% and 2.4%, respectively, from 2005 levels. Field evidence shows that local governments did manipulate emission data [63]. The centralization of emission data management was another key element for local governments’ real implementation starting from 2007, when another vertical string of environmental agencies with the main responsibility of data management was established, with the key feature that data is now controlled and verified by upper level agencies. Local EPBs only do data collection and report to regional supervision centers whose administrative ranks are higher than those of the provincial governments. The regional centers verify the reported abatement projects and audit the corresponding emission reduction data through a “cumbersome” process. Field evidence shows that the details of the data examination and verification were so sophisticated that the space for fabrication shrank significantly. Finally, the annual emission reduction of a province was certificated by MEP and was used in cadre evaluation (details see, e.g., [63]). This centralized data management guaranteed that the assessment of local implementation was based on real efforts.

#### 4.2.2. How Did Local Governments Implement Policies?

Among the many SO_2_ emitting sectors, the power sector reduced its emissions around three times the net total SO_2_ reduction, by desulfurization of big plants and small plants phase-outs. The power sector emits the most SO_2_ and the abatement capacity was huge with “low hanging fruits” [6]. It was (and still is) composed of big and small units mostly belonging to five state owned enterprises (SOE) whose leaders are another track of bureaucratic personnel and have similar promotion incentives as local main leaders, therefore negotiations between the two are not that difficult. The big units overall have limited heterogeneity and can be easily added the mature flue gas desulfurization (FGD) technology. Empirical evidences, by looking at the behavioral changes of power plants [70,71] and local governments [63,72], cross validated the implementation processes:

For desulfurization in big units, the FGD facilities were quickly installed whereas their normal operation was later gradually realized along with the policy tightening. It was a price type instrument that changed the incentive for many power plants. With a premium of 1.5 cent yuan/kWh to the feed-in tariff for electricity generated from desulfurized units, it became slightly profitable to desulfurize.

To phase out small power plant units and some other outdated enterprises was a forced process, involving local main leaders and the EPBs who coordinated among many relevant bureaus, e.g., the industrial and commercial bureau to revoke licenses, utility stations to cut the power and water supply, and the public security bureau to guarantee the enforcement (the enforcement was guaranteed by the police force). In the 12th FYP, the targeted two emissions are SO_2_ plus NO_X_. SO_2_ is to be further abated. For NO_X_, it is the power plant sector who again takes the leading role of “denitrifying”.

#### 4.2.3. Why the Efforts Are Ineffective and Inefficient in Improving Air Quality?

Reducing the total emission of SO_2_, a single primary pollutant, does not necessarily improve air quality. Elementary atmospheric chemistry shows that the transformation from primary pollutants such as SO_2_, NO_X_ and primary PM to secondary pollutants is complicated and is non-linear, as described in earlier sections. Therefore, when a single type of primary pollutant emission is reduced, air quality, as a comprehensive concept containing various primary and secondary pollutant levels, might change, but not much.

We put the comprehensive air quality aside and focus on SO_2_ pollution abatement for the moment. SO_2_ transmits within a regional scope. The jurisdictional based management cannot promote inter-regional cooperation in abatement efforts (see, e.g., [7,73]). More developed places have higher demand for SO_2_ pollution abatement but their more aggressive efforts are partially offset by the transmitted amount from their less developed neighbors. Using SO_2_ inventory data from 2006 in the Beijing-Tianjin-Hebei (BTH) region [13], show that regional management could be more effective in terms of reduction in population inhaled SO_2_ (an indicator corresponding to health risk) than jurisdictional management.

Cost saving potentials from the heterogeneity of abatement costs between territories and sectors were not exploited. For example, marginal pollutant abatement costs ($/ton) differ significantly in the cities and districts of the BTH region [15,74]. Even in the power sector with very similar technologies, marginal abatement costs are considerably different. In other more heterogeneous sectors such as industrial boilers, space for cost saving is even larger. Moreover, the effect from geographical conditions influencing how pollutants transport and transform, the marginal concentration abatement costs ($/μg·m^−3^) in these territories are also different. The administrative allocation of reduction targets among territories does not allow for some flexible market mechanisms or collaborations to jointly reduce the abatement costs.

#### 4.2.4. Special Case 2: Beijing and Surrounding Territories Fought a United Battle

To improve Beijing’s air quality for the 2008 Olympic Games was a solemn promise and was attained with a whole package of permanent and temporary actions. In Beijing, with all the regular measures mentioned previously strictly implemented, more radical measures were gradually adopted after 2005 (see, e.g., [75]). Regional transported pollution was taken into account and during the Olympics multiple measures were implemented in surrounding territories including Tianjin, Hebei, Shanxi and Inner Mongolia that can briefly be summarized as shutting down plants, restricting vehicle use, performing onboard refueling vapor recovery in gas stations, strict control of construction site dust and banning of straw burning [66]. To implement them everywhere was unrealistic, instead scientific working groups (e.g., [66,76] identified key areas and screened and prioritized measures with cost-effectiveness considerations.

The Beijing Olympics air quality management, as the very first one based on regional mechanism in China, was remarkably effective. Air quality in Beijing improved significantly during and after the Olympics, but most of the effect had faded away by the end of October 2009. Most improvements were attributable to plant closures and traffic control (see, e.g., [75]) and such measures have also been used and proved effective during the 2010 World Exposition in Shanghai, the 2010 Asian Games in Guangzhou, the 2014 APEC Conference in Beijing, and the 2015 Military Parade in Beijing. These efforts became the predecessor of the current regional management mechanism initiated in the BTH region, Pearl River Delta (PRD) and Yangtze River Delta (YRD).

### 4.3. From 2013: PM_2.5_ Crisis and then “Declaration of War against Pollution”

During January and February in 2013 severe haze covered many provinces and cities in China. The haze with its unprecedentedly high index of PM_2.5_ concentration and extremely low visibility was of worldwide concern and eventually became known as the “PM_2.5_ crisis”. Rather than an overnight shift, some preludes included: discrepancies between the monitoring data from U.S. Embassy in Beijing and Beijing EPB’s descriptive air pollution index were noticed and questioned [77], the health impacts of PM_2.5_ came to light, and the rapidly expanding power of social media in evolving the haze into a social crisis [78].

Quickly responding to the crisis, the State Council and MEP issued “Ten Actions” in June 2013 and later in September the well-known Action Plan (Table 2). Premier Li Keqiang declared in September 2013 that China is not willing to and shall not “pollute first and clean up later”, instead shall treat pollution with “iron fists” [79]. Subsequently the “iron fists” was unexceptionally used in official media and local government documents. The strong political will was further solidified in the 2014 government work report, “China shall punch hard to strengthen the prevention and control of pollution, and resolutely declare war against pollution” [80].

The Action Plan for the first time sets quantitative air quality improvement goals for key regions within a clear time limit and lists ten key actions covering all the major aspects of air quality management (Table 2). From this Action Plan various policies have emerged. Here we classify the policies into major series and outline some of the recent direct policy outcomes.

1. The “total control” of SO_2_ and NO_X_ is strengthened and accelerated: The conventional methods have been fully implemented, such as desulfurization, denitrification and dust precipitation of power plants and big industry boilers, closing small ones and phase-out of outdated industrial capacity. As vehicles are important contributors to NO_x_, the early retirement of old vehicles is also emphasized. The 12th FYP is coming to an end and its total emission control goals for major pollutants have already being achieved in advance or are about to be reached.
2. The new target responsibility agreements under the Action Plan scheme: MEP coordinated the signing of provincial action plans between central and provincial governments. Provincial action plans have similar structure compared to the nation one, including firstly the provincial air quality improvement goals and then a list of measures. These goals are not explicitly linked to veto track indicators of cadre evaluation yet, but a weighted grading system has been issued with the highest weight given to air quality goals. Serious accountability is reflected by some recent reported “arranged talks” between MEP and local leaders whose jurisdiction performed poorly in this grading system [81].
3. Unify standards, build up supporting monitoring networks and perform source apportionment analysis: A common feature of these efforts is that unification within regional scope is emphasized. Many standards are being updated, including air quality and heave pollution alerting index systems, emission standards of power plants, boilers and vehicles, fuel quality standards, technical specifications of emission monitoring and accounting. Air quality monitoring regional networks are being built. Key regions and major cities are required to establish their source inventory and perform source apportionment analysis to support further policy processes. Among the cities that have finished the analysis, results are similar with different shares among the main sources in different sectors [82].
4. Regional cooperation mechanism is taking substantial steps: Based on previous experiences, in the BTH, YRD and PRD regions, special working panels coordinate joint meetings and draft regional plans and key tasks. Taking the BTH region as an example, specific cooperation tasks being proposed and prepared include “joint emergency handling mechanism for serious pollution weather”, “couplet assistance” between Beijing, Tianjin and less developed cities in Hebei Province, “joint monitoring and enforcement” of regional pollution issues such as straw burning, fuel quality and vehicle exhausts [83].
5. Cap for coal consumption: To set a cap on annual coal consumption of 3000 Million Tons of Coal Equivalent (Mtce) by 2020 was chosen as a core strategy to address the ambient air pollution in China. The BTH, YRD and PRD regions are required to cap coal consumption by 2017. Similar to total emission control, annual coal reduction goals are subcontracted to provincial and municipal governments. “Coal to gas” in power plants was first implemented in Beijing and quickly adopted by other places. However, currently (besides Beijing) most of them have suspended or canceled the initiative, mainly because natural gas is still scarce and costly in China. Evidence show that “coal to gas” in power plants in Beijing is likely to be a net social economic loss in the current specific situation [16]. Other treatments on coal such as to reallocate high-quality coal and “ultra-low-emission technology” are initiated but without shared conclusion of their efficiency. The relative cost-effectiveness of first allocating high-quality coal to power plants instead of industrial boilers, as being implemented in some places, is also challenged [84].
6. Revising law and enhancing enforcement: A dozen of “serious cases of environmental damage” subject to criminal penalties are made explicit [85]. In 2014 more than 8000 suspects were arrested in more than 2000 environmental criminal actions, an amount which is twice the number of cases in all the previous 10 years [86]. The Environment Protection Law was amended in 2014. From the legal liability perspective, fines on illegal discharge are now imposed consecutively on a daily basis without a cap. In the first half year of 2015 after the new law coming into force, cases found applicable to several new penalty measures have been doubling and tripling on a monthly base [81]. Other specific laws on air, water and soil are under revision but the process is not as smooth as the revision of the basic law.
7. Public participation and civil society’s role is increasing: Litigation qualification for non-governmental actors is defined in the new law. Around 300 environmental “social organizations” are qualified to file litigation to the people’s courts. Information disclosure started from public available real-time air quality monitoring data, and now expands to pollution source related data such as records of enterprises’ penalties. In some provinces, the EPBs are reported as mobilizing in a campaign style to innovatively disclosure information and promote public participation in supervision and reporting of environment related illegal practices.
8. Price and quantity type market instruments are being simultaneously designed in full swing: For pollutants, pollution fees are to be changed into pollution taxes as reflected in the Environmental Protection Tax Law (draft for soliciting opinions, released in June 2015). Tradable pollutant permits are implemented in pilot provinces and cities. Primary markets are established, whereas transactions of permits in secondary markets are not frequent [87]. Carbon taxes and tradable permits, though not directly targeting air pollution, obviously are relevant. There are different tendencies between governmental departments. The National Development and Reform Commission (NDRC) has been leading the preparation of tradable carbon permits. The Ministry of Finance has been involved in designing a carbon tax. Theoretical research does not provide a clear cut conclusion on the choice of price and quantity type instrument, nor on the possibility to realize first best abatement levels when the two types coexist. Rather it is a case by case situation where different emissions coexist and abatement costs differ (see, e.g., [88]). Therefore, more empirical research is needed in the current Chinese context. Knowledge beyond economics is also important. For example, the current local tax collecting system in China may be used for collecting carbon and environmental taxes [89], whereas a tradable permits system requires new administrative departments to be set up.

## 5. Discussion

### 5.1. The Driving Factors of Policy Changes

Until now we have seen three major changes of air pollution control policies with significant implementation involving: (1) the transition in implementation of total emission control on SO_2_; (2) the temporary but effective regional air quality management at the 2008 Olympics and repeated several times later, which leads the current efforts for development of regional air quality management; and (3) the latest policy waves triggered by a PM_2.5_ crisis. These changes do not necessarily suggest that China is undergoing fundamental reform on her own initiative. Rather they, as good examples, reflect some everlasting features of Chinese governance logic. The external force driving the changes, especially the latest wave, probably can only be attributed to the profound changes in social development. Literature looking at the institutional logic of governance and the reasons for the repeated and circle governance phenomena from history to present in China has made important progress (see, e.g., [45]). We do not go deep into this line of literature but borrow some of their shared conclusions to try to explain the three policy changes.

The transition in implementation of total emission control on SO_2_ is an example of decentralization and recentralization of power, which is one theme of dynamics in the principal-agent relationship between central and local government in China. From the very beginning, air quality management, which should have been seen as a public good provision, was decentralized and administrative subcontracted together with other economic development tasks. Implementation was bound to fail due to the intrinsic problems in the principal-agent relationships. As an internal modification, with the system largely unchanged, the power to manage and verify the data was partially recentralized to the principal central government by the new data management system and then implementation was guaranteed, though full of distortions and inefficiencies.

The effective while short-lived regional air quality management during the mega events is a perfect example of “campaign style” regulation. In China, campaign style regulation exists almost universally from central to local levels, from history to the present. Setting aside its “legitimacy”, or pros and cons, it is an important supplement to China’s regular governance system [57]. When there is a special situation of need, campaign style regulations can be very effective by quickly mobilizing all kinds of capacities and if necessary, suspending all the regular activities to make sure that all the attention and resource are gathered to fulfill certain short-term goals. The temporary changes in air quality management in mega events are such a process.

Triggered by the PM_2.5_ crisis, the third ongoing change in air pollution control policies did start with obvious campaign style characteristics, such as intensive signal release of strong political will, immediate implementation of all the Olympics temporary measures in Beijing and among its neighbors [90]. However, after two years since the crisis, all kinds of new policies are still emerging and air quality management is likely to experience major changes. From what have happened it seems that this time it will not stop as a campaign style process. This reflects the fact that the space of “informal” governance has been shrinking and the governance is adapting itself to the changes in society. Irreversible trends such as information technology, increase in social mobility, and awareness of civil rights and public participation make it more and more difficult and costly to regulate in a flexible way such as to declare that “all is well” or perform “informal” measures. Governance is pushed forward to be more standardized, normalized, and legalized.

### 5.2. Macro Trends That Have Taken Shape

Changes in air quality management in China are the “tip of the iceberg” of an ongoing profound regime shift, including reforms of environmental management and social economic development patterns. Based on what is going on, some macro level trends are taking shape:

1. Strongest ever and lasting political will: Perhaps the most explicit change in air pollution control is that it has become one of the top priorities in all the efforts belonging to the construction of “Ecological Civilization”, and will probably stay so. Regardless of the naming, this state ideology is an “extremely important and urgent mission” as recently reconfirmed by the central power. A red line on environmental quality is also set by the central leadership, that air, water and soil quality in China shall only get better, not deteriorate [91]. The strong political will and red lines form the foundation of the ongoing changes.
2. Public participation and the civil society’s role are increasing: “Environment in China has severely deteriorated” has already become an irreversible public consensus. Along with the income increase and living standard improvement, individuals demand better environment quality. Environment right-defending actions, after a dozen of years of small scale but unremitting pursuit, have obtained breakthroughs recently. Environmental litigation qualification for non-governmental actors is guaranteed in the new law and this also enhances public expectations on claiming environmental rights. Governments’ attitude also changes. Local EPBs are becoming willing to disclose more information in order to promote public participation in helping enforcement.
3. Continuously increasing demand for “quantification”: Regular scientific quantification capacities such as monitoring, atmospheric modeling, source apportionment, health risk assessments have long been learned and developed by Chinese researchers while previously in most cases they were not utilized outside academia, except during the several mega events. Now there is an increasing demand to build up united quantification capacities and publicly available information platforms. Only with these can policy instruments such as performance standards, permits, taxes, damage liabilities, etc., become possible. This is to some extent the infrastructure for most of future policy designs.
4. “Top Design” (Ding Ceng She Ji) strategy integrating environment, climate, energy and regional development: Air pollution control is used as the breakthrough point of environmental protection reform, which in turn is used by the central power as a leading factor to reshape the economy and social development. The intention to integrate environment, energy and regional development issues under the climate context is well documented in the recently submitted China Intended Nationally Determined Contribution (INDC). For China’s self-interest [92,93], immediate actions such as cap on coal are being taken to ensure the achievement of the quantitative climate promises.

## 6. Conclusions

In this review we provide a comprehensive review of the Chinese air pollution situation and its related policies from the 1980s to the latest developments. We combine scientific and institutional backgrounds to provide the basis for understanding the decades’ air pollution control policy dynamics. We categorize these policies into three stages, and analyses the trends and driving factors of different policy changes. With China’s significant role in air pollution and related environment, energy and climate issues, more and more people are in need of deep and up-to-date understanding of China’s relevant policies. Thus we contribute by forming a common knowledge platform for future dialog among natural scientists, economists, political scientists and policy makers on this increasingly important topic.

We summarized the macro level trends of policy changes in Section 5.2. At the same time, many practical challenges emerge when descending to specific regulatory management designs and implementation. A typical example is the big controversy surrounding the revision of the Law on the Prevention and Control of Atmospheric Pollution (the revision was finished on the 29 August 2015, and the new law comes into force from 2016). This revision is originally aimed as an effort to solidify a new air quality management scheme in the form of a law. Given the many fundamental flaws of the largely inherited basic system and the ineffectiveness of total control policies, we end this review with an open-ended conclusion: to repair or to rebuild?

Based on the experiences of the total control policies, people learned that implementation and enforcement of environmental regulation can be well guaranteed by the cadre evaluation system and the centralized data management system. The legislation committee has the intention to solidify these total control measures into the new law, whereas most experts in environmental management, environmental economics, and environmental law in China disagree with the new law draft and propose the elimination of the total control measures. Total control is just one abatement measure and is not a quality based design. The 11th FYP experience has proved that insuring the implementation of measures does not necessarily bring air quality improvements. Experts argue to totally rebuild the law.

Most of the shared experts’ ideas are summarized in [94]. First and foremost, an ultimate subject should penetrate throughout the law rather than only being mentioned in the beginning, which is to protect air quality and public health. Then all the legislation and relevant indicators in cadre evaluation and liability should center on the quality based themes and goals. Some recent important experiences should also be more explicitly specified, not only be required or advocated, such as regional cooperation mechanisms, to unify and make standards consistent, the disclosure of quality based information and the possibility to make environmental civil rights claim based on this quality information. Experts’ ideas are not novel compared to international practice and are to some extent self-evident from the basic environmental economics and law perspective. The reason why it is still a hard choice for China to decide whether to keep walking on the total control track or to change paths is that the latter means to abandon, or at least major reform, the existing total control data accounting and verification mechanism, which already involved huge investments and personnel, and which the MEP and local governments are already familiar with.

Though it may not be realized in the near future, we have good reason to predict a revolutionary change. Accompanied by the irreversible changes in social development, the public good nature of air quality, or more generally environment quality, makes it destined to go from the original measure oriented departmental governance to public governance. The ultimate goal is supposed to be to enhance social welfare by protecting human health through improving air quality. Therefore, air quality based and health risk based management scheme is the general direction. For now, as always, the final format of the revised law might not be fundamentally critical since environmental governance in China is and probably will be a planning dominated process [6]. For the future, the world will be watching.

## Figures and Tables

**Table 1:** Examples of emission standards and air quality standards in China (1980s-current), summarized based on standards available at Ministry of Environment (MEP) website [49].

**Emission Standards of SO_2_, TSP and NO_x_ for Coal Fired Power Plants (mg/m^3^)**									
**Year ^1^**	**No. of Standard**		**SO_2_**	**TSP**	**NO_x_**				
1992	GB13223-91		- **^2^**	200–3300 **^3^**	-				
1996	GB13223-1996		**1200–2100 ^4^**	200–3300	**650**–1000				
2004	GB13223-2003		**400**–2100	**50–600**	**450–*1100*^4^**				
2012	GB13223-2011		**50–200**	**20–30**	**100–200**				
**Emission Standards of SO_2_, TSP and NO_x_ for Coal-Burning Boilers (mg/m^3^)**									
**Year**	**No. of Standard**		**SO_2_**	**TSP**	**NO_x_**				
1984	GB3841-83		-	200–600	-				
1992	GB13271-91		**1200–1800**	**100–400**	-				
2001	GB13271-2001		**900–1200**	**80–350**	-				
2014	GB13271-2014		**200–400**	**30–80**	**200–400**				
**Limits and Measurement Methods for Emissions from Light-Duty Vehicles ^5^ (g/km)**									
**Year**	**No. of Standard**	**Engine ^6^**	**CO**	**HC**	**NO_x_**	**HC + NO_x_**	**PM**		
2000	GB18352.1-2001	S	2.72	-	-	0.97	-		
		C	2.72	-	-	0.97–1.36	0.14–0.2		
2004	GB18352.2-2001	S	**2.2**	-	-	**0.5**	-		
		C	**1**	-	-	**0.7–0.9**	**0.08–0.1**		
2007	GB18352.3-2005	S	***2.3***	**0.2**	**0.15**	-	-		
		C	**0.64**	-	**0.5**	**0.56**	**0.05**		
2010	GB18352.3-2005	S	**1**	**0.1**	**0.08**	-	-		
		C	**0.5**	-	**0.25**	**0.3**	**0.025**		
2017	GB18352.5-2013	S	1	0.1	**0.06**	-	**0.0045**		
		C	0.5	-	**0.18**	**0.23**	**0.0045**		
**National Ambient Air Quality Standard (μg/m^3^, 24 h Average, Except for CO and O_3_)**									
**Year**	**No. of Standard**	**Grade**	**SO_2_**	**TSP**	**NO_2_**	**CO ^7^**	**O_3_^8^**	**PM_10_**	**PM_2.5_**
1982	GB3095-82	I	50	150	50	100	120	50	-
		II	150	300	100	100	160	150	-
		III	250	500	150	200	200	250	-
1996	GB3095-1996	I	**20**	**80**	**40**	100	120	**40**	-
		II	**60**	**200**	**40**	100	160	**100**	-
		III	**100**	**300**	**80**	200	200	**150**	-
2000	Amended GB3095-1996	I	20	80	40	100	***160***	40	-
		II	60	200	*80*	100	***200***	100	-
		III	100	300	80	200	200	150	-
2016	GB3095-2012	I	20	80	40	100	160	40	**15**
		II	60	200	**40**	100	200	**70**	**35**
**Technical Regulation on Ambient Air Quality Index (μg/m^3^, 24 h Average, Except for CO and O_3_)**									
**Year**	**No. of Standard**	**AQI ^9^**	**SO_2_**	**NO_2_**	**CO ^7^**	**O_3_^8^**	**PM_10_**	**PM_2.5_**	
2016	HJ633-2012	0	0	0	0	0	0	0	
		50	50	40	50	160	50	35	
		100	150	80	100	200	150	75	
		150	475	180	350	300	250	115	
		200	800	280	600	400	350	150	
		300	1600	565	900	800	420	250	
		400	2100	750	1200	1000	500	350	
		500	2620	940	1500	1200	600	550	

**^1^** Year: Start implementation year; **^2^** “-”: No such item in this standard; **^3^** Range: Different type/capacity/new-old power plants/boilers/engines apply to different levels in this range in the standard. **^4^** Values in bold: This indicator appears for the first time in the standard, or is tightened compared to the previous standard. Values in red, bold and italic: This indicator is loosened compared to that in the previous standard; **^5^** In this table we only show the standard for “vehicle of category 1” (6 seats or less, 2500 kg mass or less vehicles); **^6^** Engine type: C (compression ignition engine), S (spark ignition engine); **^7^** CO: mg/m^3^, one hour average; **^8^** O_3_: μg/m^3^, one hour average; **^9^** AQI: 0–50 (Good), 51–100 (Moderate), 101–150 (Unhealthy for Sensitive Groups), 151–200 (Unhealthy), 201–300 (Very Unhealthy), 301–500 (Hazardous). HC: Hydrocarbons; AQI: Air Quality Index.

**Table 2:** China National Action Plan on Air Pollution Prevention and Control (2013–2017) **^1^**.

**Air Quality Improvement Goal**
By 2017, the urban concentration of PM_10_ shall decrease by 10% compared with 2012; annual number of days with fairly good air quality will gradually increase Concentration of PM_2.5_ in the BTH, YRD and PRD regions shall respectively fall by around 25%, 20% and 15% PM_2.5_ annual concentration in Beijing shall be controlled below 60 mg/m^3^
**Ten Tasks**
Increase effort of comprehensive control and reduce emission of multi-pollutants Optimize the industrial structure, promote industrial restructure Accelerate the technology transformation, improve the innovation capability Adjust the energy structure and increase the clean energy supply Strengthen environmental thresholds and optimize industrial layout Better play the role of market mechanism and improve environmental economic policies Improve law and regulation system. Carry on supervision and management based on law Establish the regional coordination mechanism and the integrated regional environmental management Establish monitoring and warning system. Cope with pollution episodes Clarify the responsibilities of the government, enterprise and society. Mobilize public participation

**^1^** In this table we adopted the translation from Chinese by the Clean Air Alliance of China (CAAC). A full English version is available at http://sustainabletransport.org/china-releases-national-action-plan-on-air-pollution-control/.
